# The influence of comorbid depression and overweight status on peripheral inflammation and cortisol levels

**DOI:** 10.1017/S0033291721000088

**Published:** 2022-10

**Authors:** Anna P. McLaughlin, Naghmeh Nikkheslat, Caitlin Hastings, Maria A. Nettis, Melisa Kose, Courtney Worrell, Zuzanna Zajkowska, Nicole Mariani, Daniela Enache, Giulia Lombardo, Linda Pointon, NIMA Consortium, Philip Cowen, Jonathan Cavanagh, Neil Harrison, Edward Bullmore, Carmine M. Pariante, Valeria Mondelli

**Affiliations:** 1 Department of Psychological Medicine, King's College London, Institute of Psychiatry, Psychology and Neuroscience, London, UK; 2 National Institute for Health Research (NIHR) Maudsley Biomedical Research Centre, South London and Maudsley NHS Foundation Trust, King's College London, London, UK; 3 Department of Psychiatry, University of Cambridge, Cambridge, UK; 4 University Department of Psychiatry, Warneford Hospital, Oxford, UK; 5 Mental Health and Wellbeing, Sackler Institute, Neurology block, Queen Elizabeth University hospital, Glasgow, UK; 6 Division of Psychological Medicine and Clinical Sciences, Cardiff University Brain Research Imaging Centre (CUBRIC), Cardiff, UK

**Keywords:** Major depressive disorders, Overweight, Obesity, Inflammatio, C-Reactive Protein, CRP, HPA axis, Cortisoln

## Abstract

**Background:**

Depression and overweight are each associated with abnormal immune system activation. We sought to disentangle the extent to which depressive symptoms and overweight status contributed to increased inflammation and abnormal cortisol levels.

**Methods:**

Participants were recruited through the Wellcome Trust NIMA Consortium. The sample of 216 participants consisted of 69 overweight patients with depression; 35 overweight controls; 55 normal-weight patients with depression and 57 normal-weight controls. Peripheral inflammation was measured as high-sensitivity C-Reactive Protein (hsCRP) in serum. Salivary cortisol was collected at multiple points throughout the day to measure cortisol awakening response and diurnal cortisol levels.

**Results:**

Overweight patients with depression had significantly higher hsCRP compared with overweight controls (*p* = 0.042), normal-weight depressed patients (*p* < 0.001) and normal-weight controls (*p* < 0.001), after controlling for age and gender. Multivariable logistic regression showed that comorbid depression and overweight significantly increased the risk of clinically elevated hsCRP levels ⩾3 mg/L (OR 2.44, 1.28–3.94). In a separate multivariable logistic regression model, overweight status contributed most to the risk of having hsCRP levels ⩾3 mg/L (OR 1.52, 0.7–2.41), while depression also contributed a significant risk (OR 1.09, 0.27–2). There were no significant differences between groups in cortisol awakening response and diurnal cortisol levels.

**Conclusion:**

Comorbid depression and overweight status are associated with increased hsCRP, and the coexistence of these conditions amplified the risk of clinically elevated hsCRP levels. Overweight status contributed most to the risk of clinically elevated hsCRP levels, but depression also contributed to a significant risk. We observed no differences in cortisol levels between groups.

## Introduction

Rates of depression and obesity have risen dramatically in recent years, with each disorder separately posing a major health concern and economic cost to society (Abdelaal, le Roux, & Docherty, 2017; James et al., 2018; Malhi & Mann, 2018). Depression and obesity are highly comorbid disorders, and the coexistence of these conditions significantly increases the risk for developing subsequent disorders, likely due to the chronic inflammatory state that they induce (Ouakinin, Barreira, & Gois, 2018). High levels of inflammatory markers have been widely described in depression (Baumeister, Russell, Pariante, & Mondelli, 2014; Enache, Pariante, & Mondelli, 2019; Haapakoski, Mathieu, Ebmeier, Alenius, & Kivimäki, 2015; Howren, Lamkin, & Suls, 2009; Valkanova, Ebmeier, & Allan, 2013), with a recent meta-analysis finding that approximately 25% of patients with depression presented with elevated peripheral inflammation (Osimo, Baxter, Lewis, Jones, & Khandaker, 2019). However, weight gain also increases peripheral inflammation, and studies of patients with depression have found that 20% of patients to be obese and 50% to be overweight (Papakostas et al., 2005). Therefore, it is unclear whether the increased inflammation observed in patients with depression is predominantly due to the high rates of overweight and obesity in this group. As inflammation may be both a causal mechanism and potential treatment target for depressive symptoms, it is crucial to understand to what extent depression and weight gain each contribute to the increased inflammation observed in patients with depression (Ambrosio et al., 2018).

Inflammation likely plays a key role in modifying the bidirectional relationship between depression and obesity, but not all patients with depression demonstrate increased inflammation. Patients with depression who have both increased inflammation and metabolic disturbances may have a distinct ‘immuno-metabolic’ form of depression (Milaneschi, Lamers, Berk, & Penninx, 2020). This type of depression is a significant risk factor for weight gain and subsequent obesity (Hasler et al., 2004) and was specifically associated with increased peripheral levels of inflammatory markers, such as C-reactive protein (CRP) (Lamers et al., 2013). Increased CRP levels have clinical relevance for depression (Miller & Raison, 2016), as CRP ⩾3 mg/L was associated with an increased risk of developing depression later in life (Au, Smith, Gariepy, & Schmitz, 2015), and a lack of response to antidepressant medication (Chamberlain et al., 2019; Zhang et al., 2019). Conversely, reductions in body mass index (BMI) following weight loss interventions are associated with an improvement in depressive symptoms and lower CRP levels (Capuron et al., 2011; Perez-Cornago et al., 2014). These data demonstrate the crucial role of inflammation, as measured by CRP, in the interface between depression and weight gain (Ambrosio et al., 2018).

Depression with metabolic disturbances has also been associated with dysregulated hypothalamic-pituitary-adrenal (HPA) axis activity (Gold, 2015). The HPA axis produces the anti-inflammatory stress hormone cortisol, which is involved in regulating mood, metabolism, and circadian rhythms. Meta-analyses have suggested that patients with this subtype of depression have lower diurnal cortisol levels, meaning HPA axis hypoactivity (Juruena, Bocharova, Agustini, & Young, 2018 Lamers et al., 2013). In contrast, patients who exhibit the more classic symptoms of depression, such as reduced appetite and insomnia, tend to demonstrate HPA axis hyperactivity (Juruena et al., 2018; Lamers et al., 2013). This association was mechanistically supported by longitudinal studies finding that lower cortisol levels in children are associated with higher BMI at age 18 (Ruttle et al., 2014), and that blunted cortisol responses lead to further weight gain and increased inflammation (Champaneri et al., 2013). HPA axis hypoactivity may therefore be indicative of a pathophysiological process, capable of influencing mood and weight gain, which is unique to depressed patients with metabolic disturbances.

Research investigating inflammatory markers and their association with depressive symptoms in individuals with obesity is typically complicated by a high incidence of other inflammatory disorders, such as atherosclerosis, diabetes, cardiovascular disease, and hypertension (Upadhyay, Farr, Perakakis, Ghaly, & Mantzoros, 2018). Once patients have developed comorbid depression and obesity, they become more vulnerable to developing subsequent inflammatory conditions. These conditions are associated with an increased risk of recurrent depressive episodes (Nigatu, Bultmann, & Reijneveld, 2015), demonstrating the vicious cycle to which these patients may be liable. Therefore, it is vital to confirm the association between depressive symptoms and inflammation in a sample free of comorbid disorders, as the presence of other disorders may confound this relationship. It is also important to identify which subgroup of patients with depression are most vulnerable so that they can be prioritised for clinical interventions targeted at reducing their risk of developing further comorbidities.

Therefore, we investigated inflammatory mechanisms, in the form of pro-inflammatory hsCRP and anti-inflammatory cortisol, to determine how these biomarkers are associated with depression and overweight status. We also investigated whether the presence of comorbid depression and overweight increased inflammation to a clinically relevant level. Crucially, our study used a sample of patients with depression who were free of other comorbid disorders, with a comparison group of overweight controls, who were otherwise healthy.

This is the first study to explore differences in hsCRP levels and HPA axis activity in overweight individuals with and without depression, as well as normal-weight individuals with and without depression. Our cross-sectional study aimed to investigate: (1) whether overweight patients with depression had increased inflammation relative to all other groups; (2) what extent overweight status and depression status contributed to clinically elevated hsCRP levels ⩾3 mg/L; (3) whether overweight patients with depression had lower diurnal cortisol levels relative to all other groups.

## Methods

Clinical data, blood samples, and saliva samples were collected in a cross-sectional, observational design, as part of a multi-centre study investigating immune Biomarkers in Depression (BIODEP), through the Wellcome Trust Consortium for Neuroimmunology of Mood Disorders and Alzheimer's disease (NIMA). The study was approved by the Research Ethics Committee (National Research Ethics Service East of England, Cambridge Central, UK; approval number: 15/EE/0092). All procedures contributing to this work comply with the ethical standards of the relevant national and institutional committees on human experimentation and with the Helsinki Declaration of 1975, as revised in 2008. All participants provided written informed consent and underwent eligibility screening prior to taking part in any study procedures.

### Participants

Participants were aged 25–50 years inclusive and recruited at five clinical research study centres (King's College London, Oxford, Cambridge, Brighton and Glasgow) from primary and secondary NHS health services and the general population. Participants were excluded if they (1) were pregnant or breastfeeding; (2) were underweight (defined as BMI < 18); (3) were taking medication likely to compromise the interpretation of immunological data (including, but not limited to, statins, corticosteroids, antihistamines and anti-inflammatory medications); (4) met criteria for alcohol abuse, drug abuse or dependence in the last 6 months; (5) had participated in a clinical trial of an investigational drug within the last 12 months; (6) had lifetime history of any serious medical disorder likely to compromise the interpretation of immunological data; (7) had a recent infection or illness likely to compromise the interpretation of immunological data. Patients with depression were considered eligible if they met the criteria for major depressive disorder (MDD). Patients with depression were excluded if they had a lifetime history of bipolar disorder or non-affective psychosis. Healthy controls were considered eligible if they had no personal history of MDD or treatment with a monoaminergic antidepressant for depressive symptoms or any other indication, as well as no current or lifetime history of any major psychiatric disorder as defined by Diagnostic and Statistical Manual Version 5 (DSM-5).

### Sample groups

The current sample was selected from a larger sample of participants taking part in the BIODEP study, where participants were recruited based on their clinical response to antidepressants as described by our group previously (Chamberlain et al., 2019; Nikkheslat et al., 2019). For the purpose of the current study, participants from the BIODEP pool were grouped according to their BMI status (overweight participants with BMI ⩾ 25) and then categorised these participants according to the presence of depression (MDD patients *v.* healthy controls).

### Demographic and clinical measures

Age, gender, smoking status, and medical history were documented by semi-structured clinical interviews. Height and weight were measured for calculation of BMI (kg/m^2^) and overweight status was defined as BMI ⩾ 25. History and diagnosis of MDD and other psychiatric disorders were assessed with the Structured Clinical Interview for DSM-5 (Kübler, 2013). The severity of depressive symptoms was assessed using 17-item Hamilton Rating Scale for Depression (HAM-D) (Hamilton, 1960).

### High sensitivity C-Reactive Protein (hsCRP)

Peripheral inflammation was measured as serum levels of hsCRP, which has been demonstrated as a reliable biomarker of inflammation associated with MDD. Participants fasted for 8 h and abstained from strenuous exercise for 72 h prior to their blood draw, which was carried out between 8:00 and 10:00. Blood samples were collected in clotting tubes, allowed to coagulate at room temperature for 30–60 min, then centrifuged at 1600 Relative Centrifugal Force for 15 min. The serum samples were separated and transported to a central laboratory (Q2 solutions) where they were analysed on the day of collection. Samples were exposed to anti-CRP-antibodies on latex particles, and the increase in light absorption due to complex formation was used to quantify hsCRP levels, using Turbidimetry on Beckman Coulter AU analysers. Inter and intra-assay coefficient of variations were <10%. The measure of hsCRP was calculated from one blood draw taken at the time of clinical assessment, for each participant. In line with previous studies, clinically elevated hsCRP levels were defined as hsCRP ⩾3 mg/L (Miller & Raison, 2016; Pearson et al., 2003).

### Salivary cortisol

Participants were issued with the materials and instructions for collecting the saliva samples at the time of their clinical interview. Our previously published literature describes the collection procedure for saliva samples in more detail (Nikkheslat et al., 2019). Using salivette sampling devices (Sarstedt, Leicester, UK), samples were self-collected by participants at home at six-time points throughout the day; at awakening, 15, 30, and 60 min after awakening, 12:00 and at 20:00. Individuals who described problems during sample collection in the self-recorded questionnaire, or who did not respect the time-intervals required, were removed from the analysis. The current study included only participants who completed saliva sample collections accurately and who provided an adequate amount of saliva for cortisol measurement. Salivary cortisol levels were measured using a commercially available high-sensitivity salivary cortisol enzyme immunoassay kit from Salimetrics. SoftMax Pro 4.8 software was used to calculate the cortisol values, following a 4-parameter fit. The analytical sensitivity was set to 0.19 nmol/L. Inter and intra-assay coefficient of variations ranged from 8 to 10% and 6–10%, respectively. To investigate the activity and responsiveness of the HPA axis, we first compared the mean values at the various time points of salivary cortisol collection; and secondly, we calculated the area under the curve with respect to the increase (AUCi) for the cortisol awakening response using the four-time points of 0, 15, 30, and 60 min after awakening; and the area under the curve with respect to the ground (AUCg) for the diurnal cortisol using the three points: awakening, noon and 20:00. AUCg indicated the total amount of cortisol produced and overall HPA axis activity during the day. AUCi indicated the variation (either positive or negative) in cortisol concentration and thus signified the HPA axis reactivity and response to the stress of awakening. The formulas for the calculations of the AUC were derived from the trapezoidal formula introduced by Pruessner et al. (Pruessner, Kirschbaum, Meinlschmid, & Hellhammer, 2003).

### Statistical analysis

All statistical analyses were performed using R software, version 4.0.0. Data were evaluated for normality and logarithmic transformed if required. To assess group differences in demographic variables, Chi-squared or Kruskal–Wallis tests were used, as appropriate. To address the first hypothesis, group differences in logarithmic-transformed hsCRP levels were assessed using two-way analysis of covariance (ANCOVA), with group and gender as factors and age as a covariate. Pairwise comparisons were carried out using the False Discovery Rate (FDR) correction, with the alpha set at 0.05 (5%). Effect size differences in hsCRP groups relative to the normal-weight controls were calculated using Cohen's *d*. To address the second hypothesis, two models of multivariable logistic regression were used. The first assessed how much risk comorbid depression and overweight contributed to the hsCRP levels ⩾3 mg/L relative to other participant groups, with age and gender as covariates. The second assessed how much risk depression and overweight status (BMI ⩾ 25) separately contributed to hsCRP levels ⩾3 mg/L, with age and gender as covariates. To address the third hypothesis, group differences in cortisol awakening response (AUCi) and logarithmic-transformed diurnal cortisol (AUCg) were assessed using two-way ANCOVA, with adjustment for age and gender. The threshold for statistical significance of all tests was defined as two-tailed *p* ⩽ 0.05.

## Results

### Sample demographics

The demographic and clinical characteristics of the sample are presented in Table 1. Age and gender were significantly different between groups, while ethnicity and smoking status were similar across groups, in both samples. Although the proportion of smokers were not significantly different between groups, we repeated all analyses excluding smokers to determine if smoking status significantly influenced hsCRP and cortisol levels. As the direction of the results did not change for any of the relevant tests after excluding smokers, we have reported results for the full sample, with the data for non-smokers presented in the supplement. A similar number of overweight patients with depression and normal-weight patients with depression were currently taking antidepressant medication (47 patients *v.* 39 patients, *x*^2^ = 0.019, *p* = 0.889) and a two-way ANCOVA showed that both groups had a similar age of onset of depressive symptoms (*F*_(1, 109)_ = 1.617, *p* = 0.206), with adjustment for age and gender.

**Table 1.** Comparison of sample groups

	Groups				Group tests	
Variables	Overweight & depressed, *N* = 69	Overweight control, *N* = 35	Normal weight & depressed, *N* = 55	Normal weight control, *N* = 57	Statistic	*p*
Demographics						
Median age in years, (range)	38 (25–50)	38 (25–48)	32 (25–50)	30 (24–50)	*K* = 18.368	<0.001
Gender, female (%)	43 (62.3%)	17 (48.6%)	40 (72.7%)	44 (77.2%)	*x*^2^ = 9.495	0.023
BMI (range)	28.8 (25.3–47.8)	28.4 (25–35.6)	22.1 (18.1–24.9)	22.4 (18.4–24.9)	*K* = 161.87	<0.001
Ethnicity, white (%)	63 (91.3%)	29 (82.9%)	45 (81.8%)	45 (78.9%)	*x*^2^ = 4.102	0.251
Smoking status, smoker (%)	11 (15.9%)	3 (8.6%)	11 (20%)	6 (10.5%)	*x*^2^ = 3.199	0.362
Clinical						
Median HAM-D total score (range)	19 (14–31)	0 (0–5)	18 (14–26)	0 (0–7)	*K* = 162.83	<0.001
Currently on antidepressants (%)	47 (68.1%)	NA	39 (70.9%)	NA	*x*^2^ = 0.019	0.889
Mean age of depression onset ± s.d.	24.6 ± 9.8	NA	25.2 ± 9.4	NA	*F* = 1.617	0.206
hsCRP						
Median hsCRP (range)	2.2 (0.2–15.3)	1.3 (0.2–11.8)	0.7 (0.2–8.8)	0.5 (0.2–5.2)	*K* = 43.725	<0.001
Mean log hsCRP mg/L ± s.d.	0.28 ± 0.48	0.09 ± 0.43	−0.12 ± 0.42,	−0.25 ± 0.35	*F* = 16.43	<0.001
Cohen's d effect size of hsCRP *vs*. normal weight controls	1.25	0.89	0.35	-		
hsCRP ⩾3 mg/L (%)	26 (37.7%)	5 (14.3%)	6 (10.9%)	3 (5.3%)	*x*^2^ = 25.96	<0.001
Salivary cortisol						
Mean CAR AUCi (nmol min/L) ± s.d.	100.53 ± 248.99	75.79 ± 240.98	41.62 ± 240.43	86.46 ± 299.78	*F* = 0.473	0.701
Mean log AUCg (nmol h/L) ± s.d.	1.65 ± 0.18	1.66 ± 0.23	1.72 ± 0.2	1.7 ± 0.26	*F* = 1.25	0.293

BMI, body mass index; HAM-D, Hamilton Depression Rating Scale 17 for depressive symptoms; hsCRP, high sensitivity C-reactive protein; s.d., standard deviation; CAR, cortisol awakening response; AUCi, area under the curve with respect to increase; AUCg, area under the curve with respect to ground.

### High sensitivity C-Reactive Protein

**Fig. 1.**
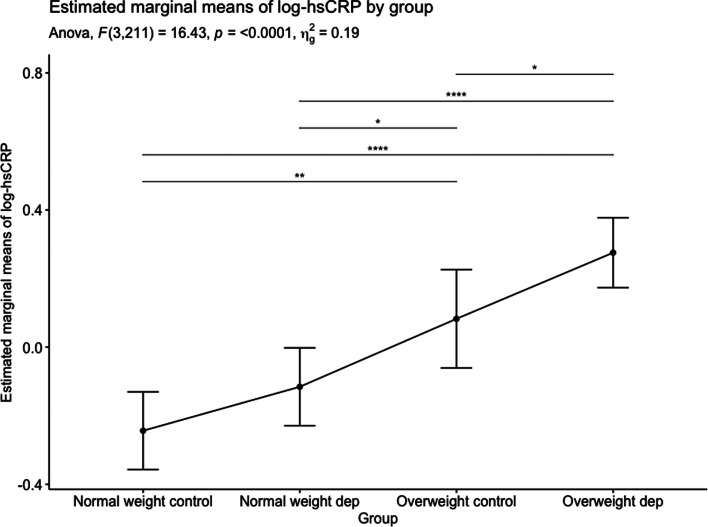
Estimated marginal means of log transformed hsCRP levels for each group. A two-way ANCOVA showed a significant effect of group on log hsCRP, with adjustment for age and gender *F*_(3, 211)_ = 16.43, *p* < 0.001. Significance of pairwise comparisons, with the False Discovery Rate correction set at 5%, are shown within the figure.

A two-way ANCOVA showed a significant effect of group on hsCRP, with adjustment for age and gender *F*_(3, 211)_ = 16.43, *p* < 0.001. As there was no significant interaction between group and gender on hsCRP, *F*_(3, 211)_ = 1.4, *p* = 0.245, pairwise comparisons with the FDR correction were carried out, comparing the main effects of group, but not gender. Overweight patients with depression had significantly higher hsCRP compared with overweight controls (*p* = 0.042), normal weight depressed patients (*p* < 0.001), and normal-weight controls (*p* < 0.001; see Fig. 1). Within the group of overweight patients with depression, there were six individuals with extreme BMI values ⩾40 indicating morbid obesity. When excluding these individuals from analysis, a two-way ANCOVA showed a significant effect of group on hsCRP, with adjustment for age and gender *F*_(3, 201)_ = 15.11, *p* < 0.001. As there was no significant interaction between group and gender on hsCRP, *F*_(3, 207)_ = 1.36, *p* = 0.257, pairwise comparisons with the FDR correction were carried out, comparing the main effects of group, but not gender. Overweight patients with depression had higher hsCRP compared with overweight controls at trend-level (*p* = 0.068), and significantly higher hsCRP compared with normal weight depressed patients (*p* < 0.001) and normal-weight controls (*p* < 0.001).

Multivariable logistic regression analysis was performed to investigate whether group status contributed to the risk of having hsCRP ⩾3 mg/L, after controlling for age and gender. Overweight patients with depression were at a significantly increased risk of having hsCRP ⩾3 mg/L [OR 2.44, 95% confidence interval (CI) 1.28 to 3.94, *p* < 0.001], overweight controls did not have a significantly increased risk of hsCRP ⩾3 mg/L (OR 1.51, 95% CI −0.33 to 2.87, *p* = 0.13), normal-weight patients with depression did not have a significantly increased risk of hsCRP ⩾3 mg/L (OR 1.08, 95% CI −0.6 to 2.4, *p* = 0.28), while normal-weight controls were at a significantly decreased risk of hsCRP ⩾3 mg/L (OR −4.39, 95% CI −7.04 to −1.97, *p* < 0.001). Age and gender were not significantly associated with hsCRP ⩾3 mg/L [OR 0.01, 95% CI −0.04 to 0.06, *p* = 0.621, OR 0.61, 95% CI −0.19 to 1.47, *p* = 0.145, respectively].

Multivariable logistic regression analysis was also performed to investigate the association between depression and overweight status on hsCRP ⩾3 mg/L, after controlling for age and gender. Depression status was significantly associated with hsCRP ⩾3 mg/L (OR 1.09, 95% CI 0.27 to 2.01, *p* = 0.013) and overweight status was significantly associated with hsCRP ⩾3 mg/L (OR 1.52, 95% CI 0.7 to 2.41, *p* < 0.001). Age and gender were not significantly associated with hsCRP ⩾3 mg/L [OR 0.62, 95% CI −0.04 to 0.06, *p* = 0.647, OR 0.01, 95% CI −0.19 to 1.47, *p* = 0.137, respectively].

### Cortisol

A two-way ANCOVA showed no significant differences between groups in CARi, after adjustment for age and gender, *F*_(3, 162)_ = 0.473, *p* = 0.701. Similarly, a two-way ANCOVA showed no significant differences between groups in logarithmic-transformed AUCg, after adjustment for age and gender, *F*_(3, 167)_ = 0.719, *p* = 0.542.

## Discussion

To our knowledge, this is the first study to observe significantly higher hsCRP levels in overweight patients with depression relative to overweight controls, as well as depressed and non-depressed normal-weight participants. Of clinical relevance, overweight patients with depression had the highest risk for hsCRP levels ⩾3 mg/L, indicating clinically elevated peripheral inflammation. Our results demonstrate that coexisting depression and overweight may exacerbate peripheral inflammation and highlights the urgent need to optimise treatment strategies for these patients. We observed no differences in HPA axis activity between groups.

Our findings regarding elevated hsCRP levels in overweight patients with depression are consistent with previous studies in normal-weight patients with depression and obese patients with depression (Haapakoski et al., 2015; Rethorst, Bernstein, & Trivedi, 2014). Of note, our study design separating overweight and normal-weight participants, from those with and without depression, was able to demonstrate the separate and comorbid association between these disorders and hsCRP levels ⩾3 mg/L. Peripheral inflammation is believed to initiate and perpetuate a range of sickness-related behaviour, such as fatigue, weakness, malaise, sleep, and disturbed appetite (Dantzer, 2006), which may further maintain the vicious cycle between weight gain and depressive symptoms. The increased risk for overweight patients with depression to have clinically elevated hsCRP ⩾3 mg/L is concerning, as CRP is a predictor of all-cause mortality (Li et al., 2017). Increased mortality and risk of developing subsequent comorbid disorders in patients with depression place a substantial burden on healthcare infrastructure and the economy (Tremmel, Gerdtham, Nilsson, & Saha, 2017), in addition to reduced quality of life for these individuals (Nigatu, Reijneveld, de Jonge, van Rossum, & Bultmann, 2016). Answering the question of whether depression or weight gain contributed more to inflammation was identified as a key objective by a recent meta-analysis (Ambrosio et al., 2018), so that research for this field can progress into therapeutic interventions designed to break the cyclic pattern between these disorders.

Testing CRP levels in overweight patients with depression could be useful for recommending adjunct anti-inflammatory treatments or predicting their likelihood of developing additional comorbidities in the future. Indeed, several clinical trials have observed that higher BMI at baseline predicted a poorer response to antidepressant treatments (Jha et al., 2018; Uher et al., 2009), while we have previously shown that immune-metabolic status was associated with a poorer response to antipsychotic medication in individuals with psychosis (Nettis et al., 2019). Anti-inflammatory drugs improve the therapeutic action of antidepressants (Bai et al., 2020; Haroon, Raison, & Miller, 2012), which could be particularly useful in the context of chronically inflamed overweight patients with depression. Overweight patients with hsCRP ⩾3 mg/L may prove ideal criterion to test this, as stratifying patients to specific treatments according to inflammatory markers seems to improve treatment response (Cuthbert & Insel, 2013; Kohler, Krogh, Mors, & Benros, 2016).

The lack of significant differences between groups in HPA axis activity was surprising, as HPA axis dysregulation has been implicated in both obesity and depression (Incollingo Rodriguez et al., 2015), however, the nature of the dysregulation is not always consistent. A systematic review comparing atypical depression with the melancholic (typical) presentation, found that atypical patients demonstrated lower cortisol relative to melancholic patients, but not so low that they were significantly different from controls (Juruena et al., 2018). In contrast, our study observed no differences in cortisol levels, despite hsCRP levels varying dramatically between groups. Dysregulation of the HPA axis may change over illness course, with hypotheses suggesting that at first cortisol levels are highly responsive to peripheral inflammation, but over time this responsivity declines (Gold, 2015; Perrin, Horowitz, Roelofs, Zunszain, & Pariante, 2019). Another explanation is that specific symptoms are responsible for changes in HPA axis activity (Iob, Kirschbaum, & Steptoe, 2020), and that antidepressant use additionally modifies HPA axis activity, as our group recently observed (Nikkheslat et al., 2019). Ultimately, our sample of patients may have been too heterogeneous in terms of depressive symptoms, antidepressant use and illness course to identify group differences.

Our study design was substantially strengthened by our strict exclusion criteria, encompassing recent illnesses and comorbid disorders, such as atherosclerosis, diabetes, and cardiovascular disease, which often confounds research in patients with obesity. We also excluded patients taking medications that could influence immunological data, such as statins, corticosteroids, antihistamines, anti-inflammatory medications Additionally, the direction of our results was maintained after we removed smokers and individuals with extreme BMI values ⩾40, suggesting that our findings are robust. Our study was limited by a lack of additional body composition and clinical measures, as these would have enabled us to define an immune-metabolic subgroup of patients within our sample. However, using overweight status as defined by BMI has high translational value for clinical settings. Our sample consisted of predominantly white participants, which limits the generalisability of the results. Our study could have been improved by the measurement of lifestyle factors, particularly physical activity, sleep and diet, as these factors may confound the relationship between depression and weight gain (Schmidt et al., 2015). Future studies should be designed to include more diverse participant samples and adjust for lifestyle factors as covariates as they may explain some of the heterogeneity in the association between depressive symptoms and inflammation.

Chronic inflammation is likely a key modifier in the vicious cycle between depressive symptoms and weight gain. If the cycle can be reversed, meaning that a reduction in depressive symptoms is accompanied by healthy behaviour such as exercise and healthy eating, which indirectly reduces inflammation and leads to excess weight loss, this would result in a better quality of life for patients and a vast reduction in global healthcare burdens and economic cost (Jantaratnotai, Mosikanon, Lee, & McIntyre, 2017; Nigatu et al., 2016; Tremmel et al., 2017). Ultimately, our data suggest that depression and overweight are separately associated with increased inflammation, but this association is amplified when these conditions coexist. Patients with depression with BMI ⩾ 25 would likely benefit from further research to determine if a higher level of clinical support or anti-inflammatory treatment approaches could potentially improve depressive symptoms and reduce the risk of developing further comorbidities in this subgroup of patients.

## Supplementary material

For supplementary material accompanying this paper visit https://doi.org/10.1017/S0033291721000088. click here to view supplementary material
