# Supplementary material for: The influence of comorbid depression and overweight status on peripheral inflammation and cortisol levels
Source: Psychol Med. 2021 Mar 18;52(14):3289–96. doi: 10.1017/S0033291721000088 (PMC9693673; doi:10.1017/S0033291721000088)
Supplement: Supplementary file 1 [file S0033291721000088sup001.docx]

**Supplementary material**

**Table 1: Comparison of sample groups with non-smoking participants only**

|  | **Groups** | | | | **Group tests** | |
| --- | --- | --- | --- | --- | --- | --- |
| **Variables** | **Overweight & depressed**  **N= 58** | **Overweight control**  **N= 32** | **Normal weight & depressed**  **N= 44** | **Normal weight control**  **N= 51** | **Statistic** | ***P*** |
| **Demographics** |  |  |  |  |  |  |
| Median age in years  (range) | 38.5  (25 – 50) | 37  (25 – 48) | 30  (25 – 50) | 30  (24 – 50) | *K* = 15.16 | 0.002 |
| Gender, female  (%) | 36  (62.1%) | 15  (46.9%) | 33  (75%) | 39  (76.5%) | *x*^2^ = 9.744 | 0.021 |
| BMI  (range) | 29.6  (25.3 – 47.8) | 28.5  (25 – 35.6) | 22  (18.1 – 24.9) | 22.4  (18.8 – 24.9) | *K* = 138.57 | <0.001 |
| Ethnicity, white  (%) | 52  (89.7%) | 26  (81.2%) | 36  (81.8%) | 40  (78.4%) | *x*^2^ = 2.711 | 0.438 |
| **Clinical** |  |  |  |  |  |  |
| Median HAM-D total score  (range) | 19  (14 – 31) | 0  (0 – 5) | 18  (14 – 26) | 0  (0 – 7) | *K* = 141.16 | <0.001 |
| Currently on antidepressants  (%) | 37  (63.8%) | NA | 31  (70.5%) | NA | *x*^2^ = 0.245 | 0.621 |
| Mean age of depression onset ± SD | 25 ± 9.8 | NA | 25.4 ± 9.2 | NA | *F* = 1.623 | 0.206 |
| **hsCRP** |  |  |  |  |  |  |
| Median hsCRP (range) | 2.25  (0.2 – 15.3) | 1.4  (0.2 – 11.8) | 0.7  (0.2 – 8.8) | 0.5  (0.2 – 5.2) | *K* = 30.514 | <0.001 |
| Mean log hsCRP mg/L ± SD  Cohen’s *d* effect size *vs* controls | 0.27 ± 0.51  1.11 | 0.09 ± 0.48  0.81 | -0.11 ± 0.43  0.29 | -0.23 ± 0.36 | *F* = 12.07 | <0.001 |
| hsCRP ≥3mg/L  (%) | 23  (39.7%) | 4  (12.5%) | 5  (11.4%) | 3  (5.9%) | *x*^2^ = 24.405 | <0.001 |
| **Salivary cortisol** |  |  |  |  |  |  |
| Mean CAR AUCi (nmol min/L) ± SD | 99.8 ± 262 | 87.7 ± 238 | 6.79 ± 228 | 89 ± 311 | *F* = 0.889 | 0.448 |
| Mean log AUCg (nmol hour/L) ± SD | 1.66 ± 0.18 | 1.67 ± 0.23 | 1.7 ± 0.18 | 1.72 ± 0.23 | *F* = 0.082 | 0.495 |

BMI= body mass index, HAM-D= Hamilton Depression Rating Scale 17 for depressive symptoms, hsCRP= high sensitivity C-reactive protein, SD = standard deviation, CAR= cortisol awakening response, AUCi= area under the curve with respect to increase, AUCg= area under the curve with respect to ground.

**NIMA (Part 1) Consortium members**

Cambridge

Edward T. Bullmore (MD, PI, EC)^1,2,11^, Junaid Bhatti^1^, Samuel J. Chamberlain^1,2^, Marta M. Correia^1,12^, Anna L. Crofts^1^, Amber Dickinson*, Andrew C. Foster*, Manfred G. Kitzbichler^1^, Clare Knight*, Mary-Ellen Lynall^1^, Christina Maurice^1^, Ciara O'Donnell^1^, Linda J. Pointon^1^, Peter St George Hyslop^1,13,14^, Lorinda Turner^31^, Petra Vertes^1^, Barry Widmer^1^, Guy B. Williams^1,14^

Cardiff

B. Paul Morgan (PI)^15^, Claire A. Leckey^15^, Angharad R. Morgan*, Caroline O'Hagan*, Samuel Touchard^15^

Glasgow

Jonathan Cavanagh (PI, EC)^3^, Catherine Deith*, Scott Farmer^16^, John McClean^16^, Alison McColl^3^, Andrew McPherson*, Paul Scouller*, Murray Sutherland^16^

Independent advisor

H.W.G.M. (Erik) Boddeke (EC)^17^

GSK

Jill C. Richardson (EC)^18^, Shahid Khan^11^, Phil Murphy^19^, Christine A. Parker^19^, Jai Patel^11^

Janssen

Declan Jones (EC)^6^, Peter de Boer^4^, John Kemp^4^, Wayne C. Drevets^6^, Jeffrey S. Nye (deceased), Gayle Wittenberg^6^, John Isaac^6^, Anindya Bhattacharya^6^, Nick Carruthers^6^, Hartmuth Kolb^6^

Kings College London

Carmine M. Pariante (PI)^10^, Federico Turkheimer (PI)^20^, Gareth J. Barker^20^, Heidi Byrom^10^, Diana Cash^20^, Annamaria Cattaneo^10^, Antony Gee^20^, Caitlin Hastings^10^, Nicole Mariani^10^, Anna McLaughlin^10^, Valeria Mondelli^10^, Maria Nettis^10^, Naghmeh Nikkheslat^10^, Karen Randall^20^, Hannah Sheridan*, Camilla Simmons^20^, Nisha Singh^20^, Victoria Van Loo*, Marta Vicente-Rodriguez^20^, Tobias C. Wood^20^, Courtney Worrell*, Zuzanna Zajkowska*

Lundbeck

Niels Plath (EC)^21^, Jan Egebjerg^21^, Hans Eriksson^21^, Francois Gastambide^21^, Karen Husted Adams^21^, Ross Jeggo*, Christian Thomsen^21^, Jan Torleif Pederson^21^, Brian Campbell*, Thomas Möller*, Bob Nelson*, Stevin Zorn*

University of Texas (sub-contracted to Lundbeck)

Jason O'Connor^22^

Oxford

Mary Jane Attenburrow (PI)^7,23^, Alison Baird, Jithen Benjamin^23^, Stuart Clare^25^, Philip Cowen^7^, I-Shu (Dante) Huang^24^, Samuel Hurley*, Helen Jones^23^, Simon Lovestone^7^,(AD, PI, EC) Francisca Mada*, Alejo Nevado-Holgado^7^, Akintayo Oladejo*, Elena Ribe^7^, Katy Smith^23^, Anviti Vyas*

Pfizer

Zoe Hughes*, Rita Balice-Gordon*, James Duerr*, Justin R. Piro*, Jonathan Sporn*

Southampton

V. Hugh Perry (PI)^27^, Madeleine Cleal*, Gemma Fryatt^27^, Diego Gomez-Nicola^27^, Renzo Mancuso^32^, Richard Reynolds^27^

Sussex

Neil A. Harrison (PI, EC)^28^, Mara Cercignani^28^, Charlotte L. Clarke^28^, Elizabeth Hoskins*, Charmaine Kohn*, Rosemary Murray*, Lauren Wilcock^29^, Dominika Wlazly^30^

University of Toronto (sub-contracted to Cambridge)

Howard Mount^13^

MD = Mood disorder workpackages lead

AD = Alzheimer’s disease workpackages lead

PI = Principal Investigator

EC = Executive committee member

^1^ Department of Psychiatry, School of Clinical Medicine, University of Cambridge, CB2 0SZ, UK

^2^ Cambridgeshire and Peterborough NHS Foundation Trust, Cambridge, CB21 5EF, UK

^3^ Sackler Centre, Institute of Health & Wellbeing, University of Glasgow, Sir Graeme Davies Building , Glasgow, G12 8TA, UK

^4^ Neuroscience, Janssen Research & Development, Janssen Pharmaceutica NV, Turnhoutseweg 30, B-2340, Beerse, Belgium

^5^ The Maurice Wohl Clinical Neuroscience Institute, Cutcombe Road, London, SE5 9RT, UK

^6^ Neuroscience, Janssen Research & Development, LLC, Titusville, NJ, 08560, USA

^7^ Department of Psychiatry, University of Oxford, Warneford Hospital, Oxford, OX3 7JX, UK

^8^ Brighton & Sussex Medical School, University of Sussex, Brighton, BN1 9RR, UK

^9^ Sussex Partnership NHS Foundation Trust, Swandean, BN13 3EP, UK

^10^ Kings College London, Institute of Psychiatry, Psychology and Neuroscience, Department of Psychological Medicine, London, SE5 9RT, UK

^11^ Immuno-Psychiatry, Immuno-Inflammation Therapeutic Area Unit, GlaxoSmithKline R&D, Stevenage SG1 2NY, UK

^12^ MRC Cognition and Brain Sciences Unit, 15 Chaucer Road, Cambridge CB2 7EF, UK

^13^ Tanz Centre for Research in Neurodegenerative Diseases, 60 Leonard Avenue, Toronto, ON M5T 2S8 Canada

^14^ Department of Clinical Neurosciences, University of Cambridge, CB2 0SZ, UK

^15^ Cardiff University, Cardiff CF10 3AT, UK

^16^ NHS Greater Glasgow and Clyde, 1055 Great Western Rd, Glasgow G12 0XH, UK

^17^ University of Groningen, 9712 CP Groningen, Netherlands

^18^ Neurosciences Virtual PoC DPU, GlaxoSmithKline R&D, Stevenage SG1 2NY, UK

^19^ Experimental Medicine Imaging, GlaxoSmithKline R&D, Stevenage SG1 2NY, UK

^20^ King's College London, Department of Neuroimaging Sciences, Institute of Psychiatry, Psychology & Neuroscience, De Crespigny Park, London SE5 8AF, UK

^21^ H. Lundbeck A/S Ottiliavej 9, 2500, Valby, Denmark

^22^ University of Texas Health Science Center at San Antonio, 7703 Floyd Curl Dr, San Antonio, TX 78229, USA

^23^ NIHR Oxford cognitive health Clinical Research Facility, Warneford Hospital, Oxford, OX3 7JX, UK

^24^ The Kennedy Institute of Rheumatology, Roosevelt Dr, Oxford OX3 7FY, UK

^25^ Oxford Centre for Functional MRI of the Brain, John Radcliffe Hospital, Oxford OX3 9DU, UK

^26^ Pfizer, Inc, 1 Portland Street, Cambridge MA, USA

^27^ Centre for Biological Sciences, University of Southampton, Southampton, UK

^28^ Clinical Imaging Sciences Centre (CISC), University of Sussex, Brighton, BN1 9RR, UK

^29^ Sussex Partnership NHS Foundation Trust, Nevill Avenue, Hove BN3 7HZ, UK

^30^ Brighton & Sussex University Hospitals NHS Trust, Brighton BN2 5BE, UK

^31^ Department of Medicine, School of Clinical Medicine, University of Cambridge, CB2 0SZ, UK

^32^ VIB-KU Leuven Center for Brain & Disease Research, Campus Gasthuisberg, Herestraat 49, bus 602, 3000 Leuven, Belgium

*Former consortium members
